# Assessment of Five Concrete Types as Candidate Shielding Materials for a Compact Radiation Source Based on the IECF

**DOI:** 10.3390/ma16072845

**Published:** 2023-04-03

**Authors:** Rawheya Ahmed, Galal Saad Hassan, Thomas Scott, Mahmoud Bakr

**Affiliations:** 1 Physics Department, Faculty of Science, Assiut University, Assiut 71516, Egypt; 2 Southwest Nuclear Hub, School of Physics, University of Bristol, Bristol BS8 1TL, UK

**Keywords:** radiation shielding materials, inertial electrostatic confinement, neutron and X-ray applications, fast neutrons removal cross-section, mass attenuation coefficient, concrete material

## Abstract

A radiation source based on the inertial electrostatic confinement fusion (IECF) system is being developed for multidisciplinary research applications. The radiation outputs from the IECF system are 2.45 MeV fast neutrons and the associated co-generated X-rays with an energy less than 3 MeV. A radiation shielding study has been performed on five types of concrete to define the most efficient material for the shielding design of the system. The proposed materials were ilmenite-magnetite concrete (IMC), ordinary concrete-1 (OC-1), barite-containing concrete (BC), ordinary concrete-2 (OC-2), and serpentine-containing concrete (SC). A numerical model was applied to determine the effective removal cross-section coefficients (∑*_Rt_*) for the fast neutrons and the total mass attenuation coefficients (*µ_m_*), the half-value layer (*HVL*), the mean free path (*MFP*), the effective atomic number (*Z_eff_*), and effective electron density (*N_eff_*) for photons inside the materials. The model considered the radiation source energy and the material properties of the concrete types. The results revealed that the serpentine-containing concrete exhibited the highest ∑*_Rt_* with 12 cm of concrete thickness needed to attenuate an incident neutron flux to 1/100 of its initial value. In addition, the BC shows the highest *µ_m_* with a 38 cm concrete thickness needed to attenuate the 3 MeV energy X-ray flux to 1/100 of its initial value. This study suggests that a 40 cm thickness of SC or BC adequately shields the radiation generated from an IECF system with a maximum particle production rate of up to 1 × 10^7^ n/s.

## 1. Introduction

Since the discovery of nuclear radiation, such as X-rays by Rontgen in 1895, γ-rays by Paul Villard in 1900, and neutrons by Chadwick in 1932 [1], X-rays, γ-rays, and neutrons have been investigated and used widely in scientific activities, as well as industrial and medical applications worldwide. The unique features of the photons and neutrons, such as the tunable range, penetration, and energy, encourage researchers to develop and optimize methods and techniques for refining their generation, and subsequently using these radiation sources for research. However, the ionizing effect of radiation makes it potentially dangerous to human health if accumulated with a significant dose [2,3]. Thus, before building any facility housing a radiation generator, it is necessary to have a design study, a risk evaluation, shielding requirements, and to quantify the expected dose exposure level around the facility [4,5,6,7]. To sufficiently reduce the intensity of the residual radiation field outside the restricted area immediately around the generator, adequate radiation shielding is mandatory to enable certifiable safe operation. The primary purpose of radiation shielding is to reduce the energy and intensity of the emitted radiation to an acceptable level outside the radiation facility. Choosing materials that efficiently attenuate the radiation is the first step of the system requirements. Secondary considerations include the volume and cost of materials, as well as how easy they are to fabricate and manipulate to form the shielding structure. The dose received outside the restricted area depends on the radiation characteristics and material properties used for the shielding [8,9,10]. Concrete and lead are the most popular shielding materials for photons (X- and γ-rays), while hydrogenic materials and light elements are adequate for neutrons [11]. Generally, the radiation types that must be considered are X- or γ-rays, and neutrons. Hence, this work focuses on studying the attenuation of these radiation types using concrete-based shielding materials containing different particulate additives. Heavy-weight concrete is used in a wide range of applications to shield radiation in fission reactors, particle accelerators, radioisotopes, fusion reactors, and sealed sources worldwide [2,12,13,14,15,16]. The selection of the shielding material depends on several factors, such as the abundance, cost, facility layout, type of radiation and energy generated [17].

A radiation facility based on an inertial electrostatic confinement fusion (IECF) system [18,19,20,21] is under construction inside a 3 × 3 × 3 m^3^ lab in the Physics Department at Assiut University. The central technology is integrated with other facilities for multidisciplinary applications, including material science, medicine, and radiography. The planned IECF system will generate 2.45-MeV neutrons and hard X-rays from the deuterium-deuterium fusion occurring inside the system, with concurrent particle scattering interactions within the system components. Determining the shielding requirement and the necessary concrete thickness to attenuate the radiation to 1/100 of its initial value is the central core of the present study. An essential parameter for radiation shielding in a material is the attenuation efficiency (% reduction in flux) compared to incident radiation. The attenuation of the neutrons in the shielding materials changes based on the neutron energy and the microstructure of the shielding material [22,23], which for thermal neutrons is referred to as the macroscopic thermal neutron cross-section attenuation. In contrast, for fast neutrons, this is referred to as the macroscopic effective removal cross-section [24,25,26]. Meanwhile, the mass attenuation coefficient is considered the primary parameter to describe the relationship between the incident photons and the shielding material [27,28,29].

Simulations using MCNP-6 and/or PHITS are planned to confirm the numerical calculations expected from the presented work to track the individual neutron and photon interactions. In the simulation, the chosen material will be investigated further in addition to the following considerations: (i) the effect of the anode material, (ii) the possibility of having a water jacket around the anode for both cooling and moderating the generated neutrons, (iii) the optimal thickness of the water jacket (0.5~7 cm) combined with concrete shielding, and (iv) modelling of a potential lead (Pb) layer after the concrete shielding to block any photons generated through the concrete by neutrons interactions. In the current work, five different types of concrete are investigated to determine their suitability as a shielding material for the proposed IECF system. The paper is arranged as follows; second Section 2 shows the radiation facility configuration and layout, the properties of the investigated concrete types, and the theoretical model used to calculate the attenuation parameters. The results and the discussion are given in Section 3. Recommendations and future work are stated in Section 4.

## 2. Materials and Methods

### 2.1. Radiation Source and the Facility Layout

The proposed radiation source in this study is a fusion system based on IECF [20]. The straightforward configuration of the system is a concentric spherical cathode connected to a negative bias, surrounded by a spherical anode that serves as a vacuum vessel. The cathode is fabricated from titanium (Ti-6Al-4V) using selective laser melting and 3D printing to form a buckyball shape with an 8 cm diameter and 0.15 cm thickness [30]. The anode is fabricated from stainless steel (SS-314) with a 40 cm inner diameter and 0.5 cm thickness [31]. The fuel in the system is deuterium (D) gas fed to the vacuum vessel through a leak valve to control the chamber gas pressure. A high-voltage power supply with a maximum capacity of 8 kW (100 kV and 80 mA) drives the system to produce up to 1 × 10^7^ n/s DD neutrons. The IECF operation requires several tens of mA current and tens of kV voltage to be applied on the cathode to establish a potential well between the cathode and anode. Thermionic electrons are generated from the cathode; these electrons ionize the D_2_ gas, start a glow discharge between the electrodes, and subsequently ‘spike’ a D plasma inside the system. The D ions in the plasma are then accelerated by the steep potential field gradient towards the centre of the cathode. Thus, fusion occurs in this central collision zone between ions and/or electrode surfaces, generating neutrons and protons, as shown in Equation (1).

D^+^ + D^+^ → ^3^He + *n* (2.45 MeV) or T + *p* (3.03 MeV) (1)

**Figure 1:**
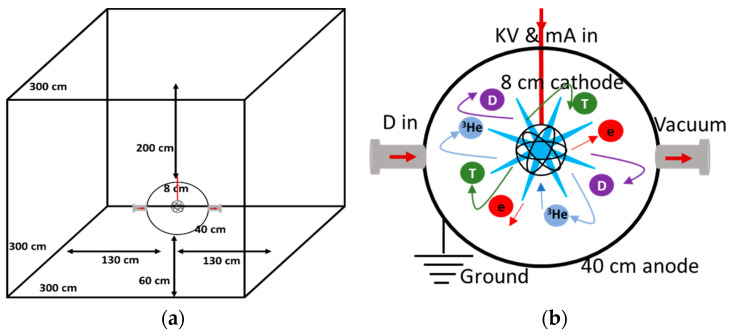
The 3 × 3 × 3 m^3^ lab layout, including the radiation source at the centre (**a**), the IECF chamber configuration system layout, and the ion traces (**b**).

Of the particles generated, only the high-energy neutrons (2.45 MeV) can escape outside the system after passing through the anode. Due to the electrical properties and the small mean free path of the generated protons, they interact with the system components and lose their energy through the collision, scattering, and release of photons with different energies. Fast neutrons with a 2.45 MeV energy and photons with energy less than 3 MeV are the main types of radiation escaping the system and hence, are the subject of this study. He and T in Equation (1) are internally consumed or collide with the system components and lose their energy. Figure 1a shows the proposed facility layout, including the IECF vacuum vessel. The output radiation from the IECF system is volumetric around the centre of the cathode [32,33]. The core of the radiation source is assembled at the centre of a cubic room of 3 × 3 × 3 m^3^, 60 cm from the floor, and 130 cm from each side with 200 cm from the top. A schematic drawing for the vacuum vessel, including the electrode dimensions, observation windows, and the fusion traces in the vessel are given in Figure 1b.

### 2.2. Shielding Materials

The most critical factor for reducing the effect of the emitted radiation is determining suitable shielding materials. Five different types of concrete with high strength and long lifetimes that are widely used in Egypt are investigated in this study. The concrete types are (1) ilmenite-magnetite concrete (IMC), (2) ordinary concrete-1 (OC-1), (3) barite-containing concrete (BC), (4) ordinary concrete-2 (OC-2), and (5) serpentine-containing concrete (SC). The chemical compositions, mass numbers, and atomic numbers of the elements, as well as the fractional weights, partial densities, and densities of the concrete materials are shown in Table 1 [6,15,34,35].

### 2.3. Calculation Model

#### 2.3.1. Neutron Attenuation

As they are electrically neutral, neutrons can only interact with the nuclei of obstructing atoms when passing through a material medium, imparting some or all of their energy. The interaction occurs here in different ways: collision or scattering, and energy absorption by the nuclei it collides with. The probability of each event depends on the neutron energy and the material properties [36]. Fast neutrons, in the MeV to eV range, attenuate primarily by losing energy through scattering interactions in the matter, with a low possibility of absorption. In addition, if the shielding materials include light atoms such as hydrogen, the neutrons slow down through a chain of elastic collisions and energy is absorbed when the energy becomes low (0.025 eV) [24]. Thermal neutrons, in the sub-meV range, are intended for absorption by heavy atoms through nuclei capture events rather than scattering in most cases. Neutron attenuation through matter can be given in the form [37,38]:

(2) $$\frac{I}{I_{0}}=\exp\;\left(-\underset{t}{\sum }x\right)$$

where *I*_0_ and *I* are the incident and attenuated neutron intensities before and after passing through the distance *x* (cm) of shielding material. The value ∑*_t_* (cm^−1^) is the physical quantity that describes the neutron attenuation in the matter, while it is called the total macroscopic cross-section, which can be given as [39,40,41]:

(3) $$\Sigma_{t}=\frac{\rho N_{a}\sigma_{t}}{A}$$

where *ρ* (g cm^−3^) is the material density, *N_a_* (Mol^−1^) is Avogadro’s number, *σ_t_* (cm^2^) is the microscopic cross-section that includes the scattering- and absorption-microscopic cross-section (*ρ*_t_ = *ρ*_s_ + *ρ*_a_), and *A* (g mol^−1^) is the atomic mass number. One can see that the value of the total macroscopic cross-section in Equation (3) depends on the material’s properties and the incident neutrons through the macroscopic cross-section, which changes based on the neutron energy. Therefore, another parameter to describe the neutron attenuation called the removal cross-section ∑*_R_* (cm^−1^), is widely used [4,42,43,44,45,46]. The ∑*_R_* is the probability that a fast or fission energy neutron takes a first collision, which removes it from the group of penetrating uncollided neutrons [47,48]. The relation between the macroscopic cross-section and the removal cross-section depends on the hydrogen percentage in the medium material and the neutron energy. When neutrons with energies between 2 and 12 MeV penetrate a medium containing a high concentration of light atoms, such as hydrogen, the value of ∑*_R_* = ∑*_t_*, while for a small fraction of hydrogen, the value of ∑*_R_* = 2/3 ∑*_t_* for energies between 6 and 8 MeV [24]. When the shielding material consists of one or more chemical compounds, it is not as simple as a pure element; the total effective removal cross-section (∑*_Rt_*) is given in the form [24]:

(4) $$\Sigma_{Rt}=\Sigma_{i}\;w_{i}\;{\left(\frac{\Sigma_{Ri}}{\rho }\;\right)}_{i}$$

where *w_i_* and *ρ_i_* (g cm^−3^) are the fraction by weight (indicated by the partial density of the elements) and the density of the medium material, respectively, and (∑*_Ri_*/*ρ*)*_i_* (cm^2^ g^−1^) is the mass removal cross-section of the *i*th constituent. The partial density of the *i*th constituent is given by *w_i_* = (*t_i_*)/(*ρ*), and (*t_i_*) is the fractional weight of the *i*th constituent. The effective removal cross-section of the proposed concrete shielding materials can be calculated from Equation (4).

#### 2.3.2. Photons Attenuation

The critical factor for evaluating the effect of photons on the shielding material is the linear attenuation coefficient *µ_l_* (cm^−1^), defined as how easily a beam of photons can penetrate 1 cm of the shielding material. From a radiation protection standpoint, the greater the attenuation exhibited for a small thickness *x* (cm) is, the better the material is at shielding the photons. So, the selection of shielding material for photons depends mainly on the attenuation properties it exhibits. The relation which describes a photon beam penetrating matter is given in a form similar to the neutron attenuation:

(5) $$I/I_{0}=\exp\left(-\mu_{l}x\right)$$

where *I*_0_ and *I* are the incident and attenuated photon intensities, respectively, the latter of which decreases exponentially with the distance inside the material *x* (cm). Another parameter linked to the material properties is introduced, called the mass attenuation coefficient *µ_m_* (cm^2^ g^−1^), which takes the density of the medium into account for photon attenuation and is given in the form:

(6) $$\mu_{m}=\frac{\mu_{l}}{\rho }$$

The mass attenuation coefficient for a medium composed of different elements can be calculated by [49,50]:

(7) $$\mu_{m}=\underset{i}{\sum }w_{i}{\left(\mu_{m}\right)}_{i}$$

where *w_i_* is the fractional weight given in Section 2.3.1, and the assumption assumes that the different elements are homogenously distributed throughout the material. In addition to the mass attenuation coefficient of photons, two essential parameters for the radiation protection field are introduced here, the half-value layer (*HVL*) and the mean free path (*MFP*). The *HVL* is defined as the thickness of the concrete specimens that will reduce the photon beam to half, while the *MFP* is the average distance travelled by photons between two events (collisions, scattering, etc.). Equations (8) and (9) give the values of the *HVL* and *MFP* based on the mass attenuation coefficient [12,16,51,52]:

(8) $$HVL=0.693/\mu_{m}\rho$$

(9) $$MFP=1/\mu_{m}\rho$$

In addition, the effective atomic number (*Z_eff_*) is equivalent to the atomic number but is used for compounds and mixtures of different materials, and this along with the effective electron density (*N_eff_*) are critical parameters for selecting the shielding materials. The effective electron density is derived from the effective atomic number and is defined as the number of electrons per unit mass. The following expressions introduce *Z_eff_* and *N_eff_* for the composite materials:

(10) $$Z_{eff}=\frac{\sum_{i}w_{i}A_{i}\mu_{m}}{\sum_{i}w_{i}\frac{A_{i}}{Z_{i}}\mu_{m}}$$

(11) $$N_{eff}=\frac{Z_{eff}N_{A}}{\sum_{i}w_{i}A_{i}}$$

where *Z_i_*, *A_i_*, and *w_i_* are the atomic number, atomic weight, and the weight fraction of element *i* in the material, respectively. The mass attenuation coefficient, linear attenuation coefficient, half-value layer, mean free path, effective atomic number, and electron density for the photons passing inside the proposed shielding materials are estimated using Equations (6)–(11) at different energies expected from the IECF: 3, 2, 1.5, 1, 0.5, 0.2, and 0.05 MeV.

#### 2.3.3. Methodology

The principle of IECF is trapping light atoms, such as deuterium (D), tritium (T), hydrogen (H), and helium (He), inside a potential well to generate subatomic particles [53]. The output from the IECF system is based on the input fuel; for D–D fusion, the system generates 2.45 MeV neutrons and 3.03 MeV protons, while for D–T fusion, the system generates 14.1 MeV neutrons, and for D–He fusion, 14.7 MeV protons are generated, etc. [53]. Therefore, designing adequate shielding for the radiation source depends on what type of fuel the system is burning.

**Figure 2:**
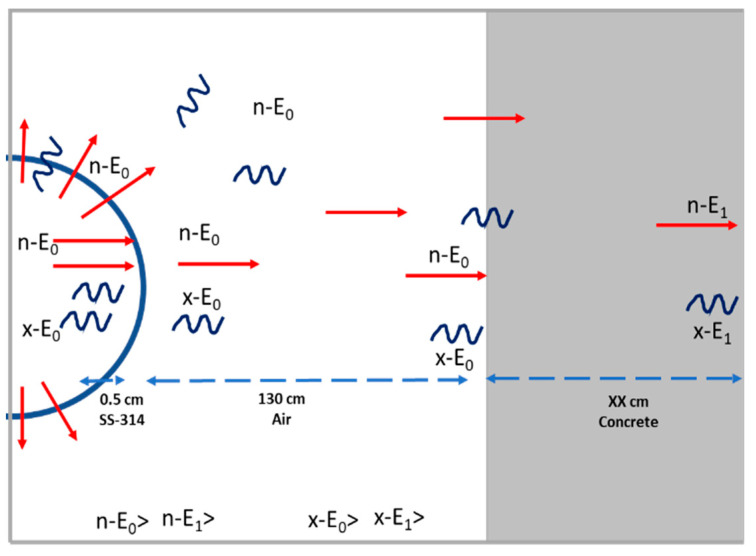
The model used to investigate the thickness of shielding material needed for the IECF system is illustrated. n–E_i_ and x–E_i_ refer to the neutron and X-ray energies generated from the IECF and attenuated through the shielding material.

In the present study, it was planned that only DD fusion takes place in the system to generate 2.45 MeV neutrons and 3.03 MeV protons. The protons have a small mean free path inside the system; they interact with the vessel materials, then lose their energy or impact with the electrode surfaces and generate photons with different energies, while the 2.45 MeV neutrons travel outside the system passing through the anode wall. Indeed, 2.45 MeV neutrons and different photon energies are generated from the system and must be shielded. In the present model, we assumed that the neutrons and photons are generated from the surface of the vacuum vessel of the system due to the fact that a large percentage of the fusion takes place at the anode surface of the IECF system [31]. The model applied in the present study to investigate the best shielding material is shown in Figure 2. In the model, 2.45 MeV neutrons and different photon energies (less than 3 MeV) are emitted from the IECF system. Then, the neutrons pass through 130 cm of the air, assuming no attenuation through the air (due to the low density of the air, 1/800 of pure water density) until reaching the shielding material. Finally, the neutrons and X-rays are attenuated through interactions with the concrete shielding. Materials that attenuate neutrons and photons over the shortest distance (thickness) to achieve a flux intensity of 1/100 of the incident radiation are considered the best for shielding applications. This study did not consider neutrons and X-ray attenuation due to scattering through the anode (0.5 cm of SS-314).

## 3. Results

### 3.1. Neutrons Attenuation

The IECF system is an isotropic radiation source, and a significant percentage of the neutrons are generated from the anode’s inner surface; therefore, we assumed that the neutrons are generated with 2.45 MeV from the outside shell of the system. The fast neutron removal cross-section of the air was ignored in this study, and neutrons travel 130 cm and reach the concrete wall without attenuation. The total microscopic cross-section values (*σ*)*_i_* of the concrete elements were collected at 2.45 MeV and then inserted into Equation (3) with the properties of the materials listed in Table 1 [54,55,56,57,58,59]. The fast neutron removal cross-section (∑*_Ri_*) values for each element of the materials were calculated, and the results are shown in Table 2. Then, the effective fast neutron removal cross-section was estimated from Equation (4) and displayed in the bottom row of Table 2. It can be seen from the table that the ∑*_Rt_* ranges between 0.09989 and 0.36635 cm*^−^*^1^.

Concrete is an engineering material that simulates rock properties and is composed of particles closely bound together using cement. From a simplistic perspective, it is a blend of aggregates, normally natural sand and gravel or crushed rock, which is held together by carbonate and calcium–silicate–hydrate (CSH), which is formed when the cement is initially wetted, and the concrete is cast into a shape. The CSH binder is the primary source of H in the concrete with regard to neutron attenuation. For the barite-containing concrete, a proportion of the aggregate used in the concrete mix is the mineral barite, also known as barium sulphate (BaSO_4_), with the Ba (*Z* = 56) adding to the overall density of the concrete. In this context, barite can be considered an additive for increasing concrete density. Serpentine-containing concrete contains serpentine minerals as a particulate additive to the aggregate. Serpentine has the generalized formula: (Mg, Fe, Ni, Mn, Zn)_2–3_(Si, Al, Fe)_2_O_5_(OH)_4_, and therefore constitutes an additive source of H in the concrete in addition to the CSH mineral content.

Based on the modelling, SC showed the highest ∑*_Rt_*, while OC-2 showed the smallest value among the five types of concrete. The high value of the ∑*_Rt_* for the SC could be attributed to the high relative ∑*_Ri_* of H (76% of ∑*_Rt_*) and the high total microscopic cross-section of the element, with high scattering and absorption cross-sections for fast neutrons in comparison to the other elements such as O (56% of the material density) in the same concrete type. On the other hand, even though OC-2 also includes H in the constituent mineral compounds, its contribution to the ∑*_Rt_* was only 17% due to the relatively small element fractional weight, 0.56% of the total density, compared to the O (50% of the material density). The effective fast neutron removal cross-section values for IMC and BC were almost the same, which could be attributed to the relatively small fractional weight of H, of less than 1% of the total density, in comparison to the high fractional weight of other elements such as O with 38 and 31% of the total density, which reduces the total microscopic cross-section (*σ*)*_i_* and hence the total effective fast neutron removal cross-section values. It is essential to mention that none of the concrete types under investigation included boron, which has a high affinity for neutron absorption. The value of ∑*_Rt_* calculated from the present methods was in good agreement with other methods presented in the literature [2,15]. As seen in Table 2, the concrete material with the highest effective fast neutron removal cross-section is the SC with ∑*_Rt_* = 0.36635 cm*^−^*^1^. This concrete type was expected to attenuate neutrons better than other concrete types.

**Figure 3:**
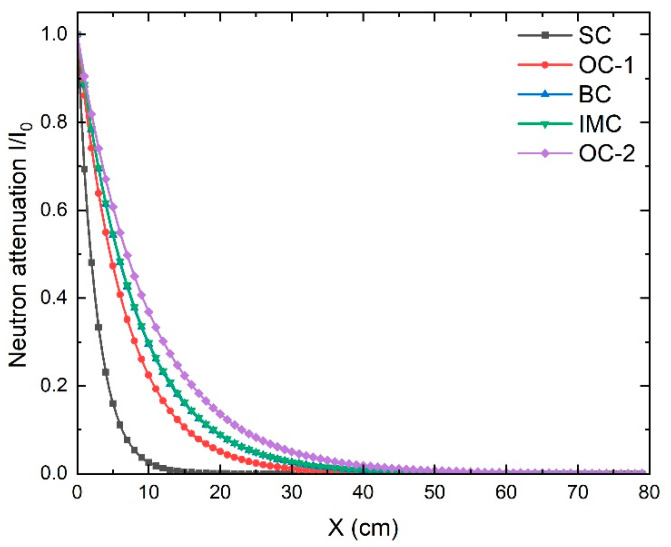
The variation in the neutron attenuation as a function of the concrete thickness for five concrete types, IMC, OC-1, BC, OC-2, and SC.

Using Equation (2), one can estimate the thickness needed to attenuate neutrons passing through the concrete materials. The effective fast neutron removal cross-section values for the five concrete types, listed in the bottom row of Table 2, were used in Equation (2) to determine the attenuation curve shown in Figure 3. The concrete thickness needed to attenuate the fast neutrons to 1/100 from the incident value was calculated from the data in Figure 3. It was found that 13, 31, 37, 38, and 46 cm were needed to attenuate the neutron intensity to 1/100 of its initial incident intensity for SC, OC-1, BC, IMC, and OC-2, respectively. Accordingly, we can conclude that serpentine concrete is the most effective concrete among the investigated types for neutron shielding. This is ascribed to it having the highest effective fast neutron removal cross-section due to the H-containing serpentine adding to the relative H-content of the concrete compared to the other candidates.

### 3.2. X-ray Attenuation

Different photon interaction processes characterize the attenuation of X- or *γ*-rays inside the shielding materials. These processes include photoelectric absorption, Compton scattering, pair production in the nuclear field, and pair production in the electric field [41,55,60,61,62,63,64]. Theoretically, the sum of these processes is represented by the total mass attenuation coefficient *µ_m_* given in Equation (5). The mass attenuation coefficients for the constituent elements of the concretes listed in Table 1 are collected to determine the total mass attenuation coefficients of the five concrete materials at different X-ray energies (3, 2, 1, 0.5, 0.2, and 0.05 MeV) generated from the IECF system [54,55,56,57,58,59].

The values of *µ_mi_* for the elemental compositions of the concrete materials for an X-ray at 1 MeV and 200 keV energies are shown in Table 3, while the *µ_mt_* values for the concrete materials for an X-ray at 1 MeV and 200 keV energies are shown in Table 4. It can be seen from the table that the values varied from 0.06113 to 0.06804 cm^2^ g*^−^*^1^, for which SC and BC exhibited the most significant and smallest total mass attenuation coefficients, respectively, among the concrete types at a 1 MeV X-ray energy. At different X-ray energies, from 0.2 MeV, the total mass attenuation coefficients for the shielding materials were calculated using the same technique, and the results are also given in Table 4. One can see that the *µ_mt_* values depend on the photon energy and the chemical composition of the concrete material. In addition, it was found that the variations in *µ_mt_* with the incident photon energy for all five concrete materials followed a similar trend. The values of *µ_mt_* decreased with the increased photon energy. Moreover, the relation between the photon energy and the *µ_mt_* values was not linear. The values of *HVL*, *MFP*, *Z_eff_*, and *N_eff_* of the photons in the shielding materials were calculated, and examples at 0.2 and 1 MeV energies are listed in Table 4. One can see that BC had the smallest *HVL* and *MFP* values; in contrast, OC-2 had the highest values. The value of *µ_mt_* calculated from the present methods is in good agreement with other techniques in the literature [49,53,54,55,56,57,58,59].

**Figure 4:**
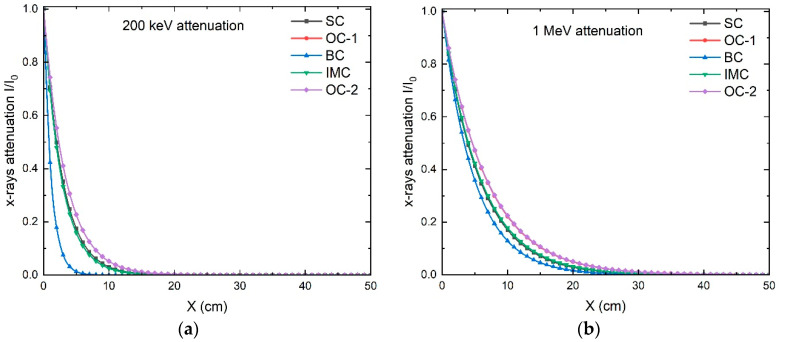
The attenuation of X-rays in the investigated concrete materials using Equation (5) at X-ray energies of (**a**) 0.2 MeV and (**b**) 1 MeV.

The X-ray attenuation curves of the concrete types were calculated using Equation (5) for all expected energies from the IECF system. The attenuation curves at 1 and 0.2 MeV X-ray energies are plotted as an example in Figure 4. It can be seen from the figure that BC was superior to the other concrete types even though SC has the largest *µ_mt_* from Table 4. This can be explained based on the effect of the concrete density, as seen in Table 1; BC has the most significant density, 3.35 g cm^−3^, compared to the other types.

**Figure 5:**
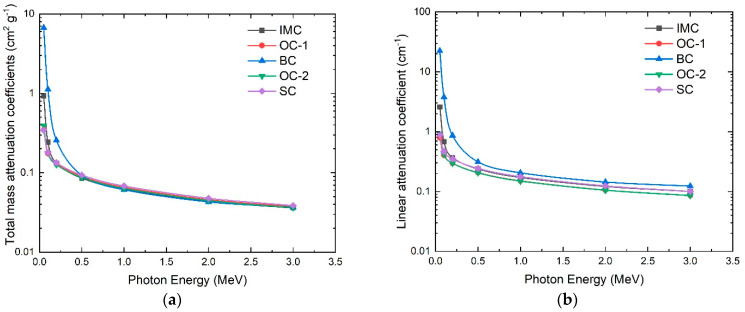

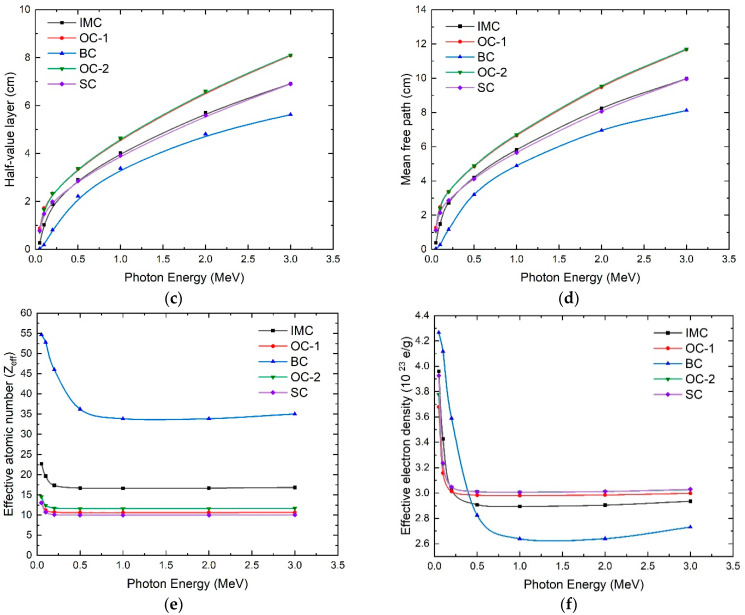
The total mass attenuation coefficients (**a**), linear attenuation coefficients (**b**), half-value layers (**c**), mean free paths (**d**), the effective atomic numbers (**e**), and effect electron densities (**f**) for X-rays with different energies from 0.05 up to 3 MeV for the investigated concrete materials.

The thickness of the shielding concrete needed to attenuate the photons to 1/100 from the initial value was calculated from the data in Figure 4. It was found that 23, 26, 27, 31, and 32 cm are needed to attenuate the 1 MeV X-ray intensity to 1/100 of its incident intensity for BC, SC, IMC, OC-1, and OC-2, respectively. In addition, 6, 13, 14, 16, and 17 cm are needed to attenuate a 0.2 MeV X-ray flux to an intensity 1/100 of its initial intensity of the BC, SC, IMC, OC-1, and OC-2, respectively. At the 3 MeV X-ray energy, the thicknesses needed to attenuate to 1/100 for BC, SC, IMC, OC-1, and OC-2 are 38, 46, 47, 54, and 55 cm, respectively. Accordingly, we could conclude that barite concrete is the most effective concrete, among the investigated types, for X-ray shielding. This was ascribed to it having the highest attenuation ratio, such that a smaller concrete thickness is required to reduce the intensity of the photons to the desired intensity. Figure 5 shows the total mass attenuation coefficients (a), linear attenuation coefficient (b), half-value layer (c), mean free path (d), effective atomic number (e), and effective electron density (f) for X-rays with different energies from 0.05 up to 3 MeV for the investigated concrete materials. It can be seen from the figure that BC had the highest *µ_mt_*, µL, *HVL*, *MFP*, and *Z_eff_* in general, and especially at low photon energy, which can be attributed to the barite atoms inside the concrete, while OC-1 and OC-2 had the smallest values. In addition to the effective attenuation of the concrete types, the price may influence the selection of the shielding material.

Before constructing the facility, MCNP-6 and/or PHITS simulations are planned to confirm the numerical calculations presented herein and track the individual neutron and photon interactions. In the simulation, (i) the effect of the anode material will be considered, (ii) the possibility of having a water jacket around the anode as a means of both cooling the anode surface and moderating the generated neutrons will be investigated, (iii) the optimal thickness of the water jacket (0.5~7 cm) combined with concrete shielding will be considered, and (iv) the modelling of a potential lead (Pb) layer after the concrete shielding to block any photons generated through the concrete through neutrons with concrete will be performed.

A final consideration of using SC for neutron shielding applications is that the precursor serpentine material would need to be elementally screened before making the concrete. This is because serpentine minerals are well documented to contain minor amounts of chromium, manganese, cobalt, or nickel, with the latter two elements providing potential routes for generating Co-60. It would be highly undesirable for the shielding material to become significantly activated (and gamma-emitting) during the lifetime of the planned facility. Further work could also explore the BC and SC formulations to further optimize the neutron and photon attenuation properties in them. It is expected that a very significant substitution of barite and serpentine for the typically used aggregate minerals in concrete (e.g., quartz) would lead to a detrimental reduction in the mechanical properties. An optimal formulation needs to be proven from both a radiation attenuation and structural performance standpoint.

## 4. Conclusions

Five concrete materials of different densities and elemental compositions were investigated in this study to assist in the selection of a shielding material for a planned radiation generator based on IECF technology. The effective removal cross-section (∑*_Rt_*) for fast neutrons and the total mass attenuation coefficients (*µ_mt_*), half-value layer (*HVL*), the mean free path (*MFP*), the effective atomic number (*Z_eff_*), and the effective electron density (*N_eff_*) for X-rays were determined for each material type and compared. The results obtained from this study show that the material density, chemical content, and total mass attenuation coefficient strongly affected the values of ∑*_Rt_* and *µ_mt_*. Serpentine concrete (SC) was found to have the highest ∑*_Rt_* value, while ordinary concrete-2 (OC-2) had the lowest value. At the same time, barite concrete (BC) and ilmenite-magnetite concrete (IMC) had almost the same values of ∑*_Rt_* for neutron attenuation. The obtained attenuation curves show that SC and BC would need the smallest material thickness to attenuate an incident neutron and photon flux to 1/100 of their initial intensities, respectively, compared to the other concrete types. The neutron attenuation curves for BC, OC-1, OC-2, and IMC were similar to that of SC. Moreover, the X-ray attenuation curves are very similar for SC, OC-1, OC-2, and IMC, with the BC being the most effective photon shield at lower incident energies.

One can conclude from this work that serpentine concrete is a good material for neutron shielding applications, whilst barite concrete is slightly better than other concrete types for shielding X-rays. In addition, 40 cm of serpentine or barite concrete is deemed sufficient to attenuate the neutrons and photons generated from the planned IECF system to 1/100 of their initial intensities. Further, more advanced simulations are needed using MCNP and PHITS to confirm the calculated parameters before constructing the facility.

## Figures and Tables

**Table 1:** The elemental composition, atomic and mass numbers, fraction by weight, partial density, and density of the concrete materials used in the present study.

Element Properties			IMC		OC-1		BC		OC-2		SC	
S	A	Z	w_i_	*ρ*_i_	w_i_	*ρ*_i_	w_i_	*ρ*_i_	w_i_	*ρ*_i_	w_i_	*ρ*_i_
H	1	1.008	0.57	0.0157	2.21	0.0508	0.36	0.0121	0.56	0.0132	7.20	0.1872
C	6	12.011	0.08	0.0022	0.25	0.0058					0.15	0.0039
O	8	15.999	37.85	1.0523	57.75	1.3283	31.18	1.0445	49.56	1.1647	55.6	1.4456
Na	11	22.989			1.52	0.0349			1.71	0.0402		
Mg	12	24.305	3.65	0.1014	0.13	0.0029	0.11	0.0037	0.24	0.0056	10.20	0.2652
Al	13	26.982	1.79	0.0497	2.10	0.0483	0.42	0.0141	4.56	0.1072	2.50	0.065
Si	14	28.086	4.85	0.1349	30.56	0.7029	1.04	0.0348	31.35	0.7367	17.55	0.4563
P	15	30.974	0.01	0.0002								
S	16	32.060	0.06	0.0016			10.78	0.3611	0.12	0.0028		
K	19	39.098			1.08	0.0248			1.92	0.0451	0.08	0.0021
Ca	20	40.080	8.88	0.2469	4.39	0.1009	5.02	0.1682	8.26	0.1941	5.64	0.1466
Ti	22	47.880	12.82	0.3563								
V	23	50.942	0.08	0.0021								
Cr	24	51.996	0.04	0.0010								
Mn	25	54.938	0.30	0.0084								
Fe	26	55.847	28.28	0.7863	0.70	0.0161	4.75	0.1595	1.22	0.0287	1.08	0.0281
Ni	28	58.690	0.04	0.0012								
Ba	56	137.330					46.34	1.5524				
**Density (g cm^−3^)**			**2.78**		**2.30**		**3.35**		**2.35**		**2.60**	

**Table 2:** The ∑*_Ri_* and the ∑*_Rt_* values for each element of the concrete materials whole material were calculated using Equations (3) and (4).

Elements	IMC	OC-1	BC	OC-2	SC
	∑*_Ri_*(cm^−1^)				
H	0.02450	0.07932	0.01882	0.02054	0.29213
C	0.00017	0.00046			0.00031
O	0.01904	0.02404	0.01890	0.02108	0.02616
Na		0.00302		0.00347	
Mg	0.00521	0.00015	0.00019	0.00029	0.01364
Al	0.00298	0.00290	0.00084	0.00642	0.00390
Si	0.00620	0.03228	0.00160	0.03384	0.02096
P	0.00001				
S	0.00009		0.02091	0.00016	
K		0.00130		0.00237	0.00011
Ca	0.01324	0.00542	0.00902	0.01041	0.00787
Ti	0.01288				
V	0.00010				
Cr	0.00005				
Mn	0.00032				
Fe	0.03601	0.00074	0.00729	0.00131	0.00129
Ni	0.00015				
Ba			0.04454		
**∑*_Rt_* (cm^−1^)**	**0.12095**	**0.14962**	**0.12211**	**0.09989**	**0.36635**

**Table 3:** The mass attenuation coefficients (*µ_ml_*) of the elements of the concrete types for 1 and 0.2 MeV X-rays.

Element	*µ_mi_* (1 MeV) (cm^2^ g^−1^)	*µ_mi_* (0.2 MeV) (cm^2^ g^−1^)
H	0.12630	0.24290
C	0.06361	0.12290
O	0.06372	0.12370
Na	0.06100	0.11990
Mg	0.06296	0.12450
Al	0.06146	0.12230
Si	0.06361	0.12750
P	0.06182	0.12500
S	0.06373	0.13020
K	0.06216	0.13190
Ca	0.06388	0.13760
Ti	0.05891	0.13140
V	0.05794	0.13180
Cr	0.05930	0.13780
Mn	0.05852	0.13910
Fe	0.05995	0.14600
Ni	0.06160	0.11990
Ba	0.05803	0.40450

**Table 4:** The total mass attenuation coefficients (*µ_mt_*), the half value layer (*HVL*), the mean free path (*MFP*), the effective atomic number (*Zeff*), and the electron density (*Neff*) of the concrete types at 0.2 MeV ^#^ and 1 MeV * X-ray energies.

Concrete Type	Total *µ_mt_* * (cm^2^ g^−1^)	*HVL* * (cm)	*MFP* * (cm)	*Z_eff_* *	*N_eff_* * (10^23^ e/g)	Total *µ_mt_* ^#^ (cm^2^ g^−1^)	*HVL* ^#^ (cm)	*MFP* ^#^ (cm)	*Z_eff_* ^#^	*N_eff_* ^#^ (10^23^ e/g)
IMC	0.06185	4.03013	5.81548	16.54016	2.89570	0.13228	1.88443	2.71924	17.35705	3.02560
OC-1	0.06538	4.60831	6.64980	10.58819	2.98000	0.12639	2.33360	3.36738	10.70426	3.01267
BC	0.06113	3.38418	4.88338	33.81705	2.63872	0.25674	0.80573	3.36738	45.99331	3.23884
OC-2	0.06350	4.64376	6.70095	11.58687	3.00708	0.12912	2.33304	3.36658	11.74539	3.04822
SC	0.06804	3.91742	5.65284	9.964079	3.00542	0.13403	1.98871	2.86971	10.10404	3.04764

## Data Availability

Data are available upon request officially from the editor.

## References

[1] Chadwick J. (1932). Possible Existence of Neutron. Nature.

[2] El Abd A., Mesbah G., Mohammed N.M.A., Ellithi A. (2017). A Simple Method for Determining the Effective Removal Cross Section for Fast Neutrons. J. Radiat. Nucl. Appl..

[3] Aloraini D.A., Sayyed M.I., Jecong J.F.M., Kumar A., Elbashir B.O., Almuqrin A.A.H., Yasmin S. (2022). Structural and Gamma Ray Shielding Behavior of Dual Heavy Metal Oxide Doped Magnesium Sodium Borate Glasses. Optik.

[4] Mostafa A.M.A., Issa S.A.M., Sayyed M.I. (2017). Gamma Ray Shielding Properties of PbO-B2O3-P2O5 Doped with WO3. J. Alloys Compd..

[5] Coleman A.C., Yukihara E.G. (2018). On the Validity and Accuracy of the Initial Rise, Method Investigated Using Realistically Simulated Thermoluminescence Curves. Radiat. Meas..

[6] Bashter I.I. (1997). Calculation of Radiation Attenuation Coefficients for Shielding Concretes. Ann. Nucl. Energy.

[7] Aloraini D.A., Sayyed M.I., Almuqrin A.A.H., Kumar A., Khazaalah T.H., Yasmin S., Khandaker M.U., Baki S.O. (2022). Preparation, Radiation Shielding and Mechanical Characterization of PbO–TeO_2_–MgO–Na_2_O–B_2_O_3_ Glasses. Radiat. Phys. Chem..

[8] Lakshminarayana G., Kumar A., Tekin H.O., Issa S.A.M., Al-Buriahi M.S., Dong M.G., Lee D.E., Yoon J., Park T. (2021). In-Depth Survey of Nuclear Radiation Attenuation Efficacies for High Density Bismuth Lead Borate Glass System. Results Phys..

[9] Kaçal M.R., Akman F., Sayyed M.I. (2019). Evaluation of Gamma-Ray and Neutron Attenuation Properties of Some Polymers. Nucl. Eng. Technol..

[10] Hassan H.E., Badran H.M., Aydarous A., Sharshar T. (2015). Studying the Effect of Nano Lead Compounds Additives on the Concrete Shielding Properties for γ-Rays. Nucl. Instrum. Methods Phys. Res. Sect. B Beam Interact. Mater. Atoms.

[11] Abdullah Aloraini D., Sayyed M.I., Kumar A., Yasmin S., Almuqrin A.H., Tishkevich D.I., Trukhanov A.V. (2022). Studies of Physical, Optical, and Radiation Shielding Properties of Bi2O3-TeO2-MgO-Na2O-B2O3 Glass System. Optik (Stuttg).

[12] El-Samrah M.G., Abreu Zamora M.A., Novog D.R., Chidiac S.E. (2022). Radiation Shielding Properties of Modified Concrete Mixes and Their Suitability in Dry Storage Cask. Prog. Nucl. Energy.

[13] Alharshan G.A., Alrowaili Z.A., Mahmoud Z.M.M., Olarinoye I.O., Al-Buriahi M.S. (2022). Effect of Nb_2_O_5_ Inclusion on the Radiation Shielding Efficiency of TeO_2_–ZnO–LiF–NaF Glass System. Radiat. Phys. Chem..

[14] Hadad K., Majidi H., Sarshough S. (2015). Enhanced Radiation Shielding with Galena Concrete. Nucl. Technol. Radiat. Prot..

[15] El-Khayatt A.M.M., El-Sayed Abdo A. (2009). MERCSF-N: A Program for the Calculation of Fast Neutron Removal Cross Sections in Composite Shields. Ann. Nucl. Energy.

[16] Waly E.-S.A., Bourham M.A. (2015). Comparative Study of Different Concrete Composition as Gamma-Ray Shielding Materials. Ann. Nucl. Energy.

[17] Gurler O., Akar Tarim U. (2012). An Investigation on Determination of Attenuation Coefficients for Gamma-Rays by Monte Carlo Method. J. Radioanal. Nucl. Chem..

[18] Hirsch R.L. (1967). Inertial-Electrostatic Confinement of Ionized Fusion Gases. J. Appl. Phys..

[19] Bakr M., Masuda K., Yoshida M. (2019). Development of a Portable Neutron Generator Based on Inertial Electrostatic Confinement D-D Fusion Reaction. Proceedings of the AIP Conference Proceedings.

[20] Bakr M., Mukai K., Masuda K., Yagi J., Konishi S. (2021). Characterization of an Ultra-Compact Neutron Source Based on an IEC Fusion Device and Its Prospective Applications in Radiography. Fusion Eng. Des..

[21] Masuda K., Kashima R., Bakr M.A. (2019). Potential Profile Measurements Inside a Gridded Cathode at High Potential in a Spherical Inertial Electrostatic Confinement Device. Fusion Sci. Technol..

[22] Almuqrin A.H., Jecong J.F.M., Hila F.C., Balderas C.V., Sayyed M.I. (2021). Radiation Shielding Properties of Selected Alloys Using EPICS2017 Data Library. Prog. Nucl. Energy.

[23] Evans B.R., Lian J., Ji W. (2018). Evaluation of Shielding Performance for Newly Developed Composite Materials. Ann. Nucl. Energy.

[24] Elmahroug Y., Tellili B., Souga C. (2014). Determination of Shielding Parameters for Different Types of Resins. Ann. Nucl. Energy.

[25] Dong M., Xue X., Liu S., Yang H., Li Z., Sayyed M.I., Agar O. (2019). Using Iron Concentrate in Liaoning Province, China, to Prepare Material for X-Ray Shielding. J. Clean. Prod..

[26] Morgan K.Z. (1980). Radiation Shielding and Dosimetry. Nucl. Sci. Eng..

[27] Awadallah M.I., Imran M.M.A. (2007). Experimental Investigation of γ-Ray Attenuation in Jordanian Building Materials Using HPGe-Spectrometer. J. Environ. Radioact..

[28] Gerward L., Guilbert N., Bjorn Jensen K., Levring H. (2001). X-Ray Absorption in Matter. Reengineering XCOM. Radiat. Phys. Chem..

[29] Liu Y., Liu B., Gu Y., Wang S., Li M. (2022). Gamma Radiation Shielding Property of Continuous Fiber Reinforced Epoxy Matrix Composite Containing Functional Filler Using Monte Carlo Simulation. Nucl. Mater. Energy.

[30] Bakr M., Wulfkühler J.-P.P., Mukai K., Masuda K., Tajmar M., Konishi S. (2021). Evaluation of 3D Printed Buckyball-Shaped Cathodes of Titanium and Stainless-Steel for IEC Fusion System. Phys. Plasmas.

[31] Bakr M., Sakabe T., Wulfkühler J.-P., Mukai K., Smith T.W., Ogino O., Tajmar M., Scott T., Konishi S. (2023). Influence of electrodes’ geometrical properties on the neutron production rate of a discharge fusion neutron source. Phys. Plasmas.

[32] Mukai K., Kenjo S., Iwamatsu N., Bakr M., Chikada T., Yagi J., Konishi S. (2022). Hydrogen Permeation from F82H Wall of Ceramic Breeder Pebble Bed: The Effect of Surface Corrosion. Int. J. Hydrogen Energy.

[33] Donovan D.C., Boris D.R., Kulcinski G.L., Santarius J.F. (2017). Fusion Science and Technology Optimization of an IEC Fusion Device to Increase Steady-State D-D Neutron Generation Rates Optimization of an IEC Fusion Device to Increase Steady-State D-D Neutron Generation Rates. Fusion Sci. Technol..

[34] El-Khayatt A.M. (2010). Calculation of Fast Neutron Removal Cross-Sections for Some Compounds and Materials. Ann. Nucl. Energy.

[35] Issa S.A.M., Saddeek Y.B., Tekin H.O., Sayyed M.I., Shaaban K. (2018). Saber Investigations of Radiation Shielding Using Monte Carlo Method and Elastic Properties of PbO-SiO2-B2O3-Na2O Glasses. Curr. Appl. Phys..

[36] Dong M.G., Xue X.X., Elmahroug Y., Sayyed M.I., Zaid M.H.M. (2019). Investigation of Shielding Parameters of Some Boron Containing Resources for Gamma Ray and Fast Neutron. Results Phys..

[37] Schad J., Rost H. (2013). Radiation Shielding. Kunststoffe Int..

[38] Korkut T., Gence O., Kam E., Brostow W. (2013). X-Ray, Gamma, and Neutron Radiation Tests on Epoxy-Ferrochromium Slag Composites by Experiments and Monte Carlo Simulations. Int. J. Polym. Anal. Charact..

[39] Seltzer S.M. (1993). Calculation of Photon Mass Energy-Transfer and Mass Energy-Absorption Coefficients. Radiat. Res..

[40] Korkut T., Karabulut A., Budak G., Aygün B., Gencel O., Hançerlioğulları A. (2012). Investigation of Neutron Shielding Properties Depending on Number of Boron Atoms for Colemanite, Ulexite and Tincal Ores by Experiments and FLUKA Monte Carlo Simulations. Appl. Radiat. Isot..

[41] Tellili B., Elmahroug Y., Souga C. (2017). Investigation on Radiation Shielding Parameters of Cerrobend Alloys. Nucl. Eng. Technol..

[42] El-Khayatt A.M., Akkurt I. (2013). Photon Interaction, Energy Absorption and Neutron Removal Cross Section of Concrete Including Marble. Ann. Nucl. Energy.

[43] Oto B., Madak Z., Kavaz E., Yaltay N. (2019). Nuclear Radiation Shielding and Mechanical Properties of Colemanite Mineral Doped Concretes. Radiat. Eff. Defects Solids.

[44] El-Sayed Abdo A. (2002). Calculation of the Cross-Sections for Fast Neutrons and Gamma-Rays in Concrete Shields. Ann. Nucl. Energy.

[45] Pagonis V., Shannon C. (2000). Improved Experimental Procedure of Separating a Composite Thermoluminescence Glow Curve into Its Components. Radiat. Meas..

[46] Atasoy H., Tarcan G., Dökmen S. (1992). Investigation of Turkish Marbles as Shielding Materials. Nucl. Inst. Methods Phys. Res. B.

[47] Sukegawa A.M., Anayama Y., Okuno K., Sakurai S., Kaminaga A. (2011). Flexible Heat Resistant Neutron Shielding Resin. J. Nucl. Mater..

[48] Hubbell J.H. (1982). Photon Mass Attenuation and Energy-Absorption Coefficients. Int. J. Appl. Radiat. Isot..

[49] Içelli O., Yalçin Z., Okutan M., Boncukçuoǧlu R., Şen A. (2011). The Determination of the Total Mass Attenuation Coefficients and the Effective Atomic Numbers for Concentrated Colemanite and Emet Colemanite Clay. Ann. Nucl. Energy.

[50] Gaikwad D.K., Obaid S.S., Sayyed M.I., Bhosale R.R., Awasarmol V.V., Kumar A., Shirsat M.D., Pawar P.P. (2018). Comparative Study of Gamma Ray Shielding Competence of WO_3_-TeO_2_-PbO Glass System to Different Glasses and Concretes. Mater. Chem. Phys..

[51] Levet A., Kavaz E., Özdemir Y. (2020). An Experimental Study on the Investigation of Nuclear Radiation Shielding Characteristics in Iron-Boron Alloys. J. Alloys Compd..

[52] Mukai K., Ogino Y., Kobayashi M.I., Bakr M., Yagi J., Ogawa K., Isobe M., Konishi S. (2021). Evaluation of Tritium Production Rate in a Blanket Mock-up Using a Compact Fusion Neutron Source. Nucl. Fusion.

[53] Michalak M.K., Fancher A.N., Kulcinski G.L., Santarius J.F. (2017). Expanding Operational Space in Inertial Electrostatic Confinement D-D Neutron Generators. Fusion Sci. Technol..

[54] El-Samrah M.G., El-Mohandes A.M., El-Khayatt A.M., Chidiac S.E. (2021). MRCsC: A User-Friendly Software for Predicting Shielding Effectiveness against Fast Neutrons. Radiat. Phys. Chem..

[55] Abo-El-Enein S.A., El-Sayed H.A., Ali A.H., Mohammed Y.T., Khater H.M., Ouda A.S. (2014). Physico-Mechanical Properties of High-Performance Concrete Using Different Aggregates in Presence of Silica Fume. HBRC J..

[56] Sigma Periodic Table Browse. https://www.nndc.bnl.gov/sigma/index.jsp.

[57] Al-Hamarneh I.F. (2017). Investigation of Gamma-Ray Shielding Effectiveness of Natural Marble Used for External Wall Cladding of Buildings in Riyadh, Saudi Arabia. Results Phys..

[58] Yasaka P., Pattanaboonmee N., Kim H.J.J., Limkitjaroenporn P., Kaewkhao J. (2014). Gamma Radiation Shielding and Optical Properties Measurements of Zinc Bismuth Borate Glasses. Ann. Nucl. Energy.

[59] Obaid S.S., Sayyed M.I.I., Gaikwad D.K.K., Pawar P.P.P. (2018). Attenuation Coefficients and Exposure Buildup Factor of Some Rocks for Gamma Ray Shielding Applications. Radiat. Phys. Chem..

[60] Oto B., Gür A., Kavaz E., Çakır T., Yaltay N. (2016). Determination of Gamma and Fast Neutron Shielding Parameters of Magnetite Concretes. Prog. Nucl. Energy.

[61] Tekin H.O., Altunsoy E.E., Kavaz E., Sayyed M.I., Agar O., Kamislioglu M. (2019). Photon and Neutron Shielding Performance of Boron Phosphate Glasses for Diagnostic Radiology Facilities. Results Phys..

[62] Sayyed M.I. (2016). Investigation of Shielding Parameters for Smart Polymers. Chin. J. Phys..

[63] Sayyed M.I. (2017). Half Value Layer, Mean Free Path and Exposure Buildup Factor for Tellurite Glasses with Different Oxide Compositions. J. Alloys Compd..

[64] D’Souza A.N., Sharmila K., Sayyed M.I., Somshekarappa H.M., Khandaker M.U., Bradley D.A., Kamath S.D. (2021). Gamma Ray Shielding and Thermoluminescence Investigation of Bismuth Added Heavy Metal Oxide Glasses. Radiat. Phys. Chem..

